# Diagnostic and Predictive Values of Circulating Extracellular Vesicle-Carried microRNAs in Ischemic Heart Disease Patients With Type 2 Diabetes Mellitus

**DOI:** 10.3389/fcvm.2022.813310

**Published:** 2022-02-28

**Authors:** Li Zhang, Jianchao Zhang, Zhen Qin, Na Liu, Zenglei Zhang, Yongzheng Lu, Yanyan Xu, Jinying Zhang, Junnan Tang

**Affiliations:** ^1^Department of Cardiology, The First Affiliated Hospital of Zhengzhou University, Zhengzhou, China; ^2^Henan Province Key Laboratory of Cardiac Injury and Repair, Zhengzhou, China; ^3^Henan Province Clinical Research Center for Cardiovascular Diseases, Zhengzhou, China; ^4^Department of Pediatrics, The First Affiliated Hospital of Zhengzhou University, Zhengzhou, China

**Keywords:** diagnostic, predictive, extracellular vesicle, ischemic heart disease, type 2 diabetes mellitus

## Abstract

Ischemic heart disease patients with diabetes mellitus (IHD-DM) have a higher risk of cardiovascular events than those without DM. Rapid identification of IHD-DM can enable early access to medical treatment and reduce the occurrence of cardiovascular adverse events. In the present study, we identified and examined extracellular vesicle (EV)-carried microRNAs (miRNAs) as the possible diagnostic biomarkers of IHD-DM. Small RNA sequencing was performed to analyze the EV-carried miRNAs spectrum, and differentially expressed miRNAs were further confirmed by quantitative real-time polymerase chain reaction (qRT-PCR). Through small RNA sequencing, we identified 138 differentially expressed EV-carried miRNAs between IHD-DM patients and healthy controls. Furthermore, we identified that five EV-carried miRNAs (miR-15a-3p, miR-18a-5p, miR-133a-3p, miR-155-5p, and miR-210-3p) were significantly down-regulated and one (miR-19a-3p) was significantly up-regulated in the IHD-DM patients compared to healthy controls. The receiver–operating characteristic curve analysis showed that the above six EV-carried miRNAs have excellent diagnostic efficacy of IHD-DM. Our findings indicated that the circulating EV-miRNAs might be promising biomarkers for the convenient and rapid diagnosis of IHD-DM.

## Introduction

Cardiovascular disease remains an important cause of death both globally and in China, as it accounts for about 40% of the causes of death in the Chinese population (1). Moreover, patients with ischemic heart disease (IHD) are often have diabetes mellitus (DM) as well, a condition referred to as IHD-DM (2). EUROASPIRE IV study (3) showed that about 27% of IHD patients had DM, and among remaining IHD patients with no reported history of DM, screening according to the WHO criteria for fasting plasma glucose (FPG) plus 2-h post-load plasma glucose (2hPG) identifies 45.7% as having a high risk for DM and 27.7% as having newly detected DM. Additionally, clinical data from China also showed that more than 50% of IHD patients suffer from DM (4). A large number of studies have shown that patients with DM are at higher risk of death than the non-diabetic population (5). Compared with IHD patients without diabetes, the risk of death in IHD patients with diabetes is also significantly increased (6). Furthermore, a recent study suggests that patients with minimal ischemia, once DM is established, have a comparable risk of major cardiovascular adverse events (MACEs) as patients with severe ischemia but not complicated with DM (7). The previous study has revealed that type 2 diabetes mellitus (T2DM) plays a vital role in accelerating the formation and rupture of atherosclerotic plaque, promoting myocardial fibrosis, and reducing cardiac function (8). Thus, establishing a method for the timely, convenient, and rapid diagnosis of IHD-DM and revealing the underlying molecular mechanism of DM promoting the pathological progression of cardiovascular disease will help to alleviate the cardiovascular damage caused by DM (9).

Secreted by a variety of cell types, extracellular vesicles (EVs) contain various proteins and RNA species. EVs play an important role in multiple physiological and pathological processes (10). As key components of EVs cargo, small non-coding RNA, especially microRNAs (miRNAs), are well-known to play a crucial role in the regulation of multiple biological effects including cell proliferation and apoptosis, fibrosis, inflammation, and angiogenesis (11). Moreover, the numbers and contents of EVs vary in different organs and pathological conditions. Thus, EV-carried miRNAs are considered to reflect the physiological and pathological alterations (12). Subsequent studies have also demonstrated that the dysregulation of EV-carried miRNAs could not only reflect the state of the disease but also participate in the pathological activities of the disease (13).

Although a large body of literature has explored the circulating miRNAs signatures in patients with T2DM or IHD (14), few studies have reported the unique expression profiles and signatures of EV-carried miRNAs in IHD-DM patients. Compared to circulating non-EV miRNAs, EV-carried miRNAs appear to be more stable and more sensitive to changes in the state of the disease (15, 16), so it is considered to be a better source for biomarker studies (17). Hence, in the present study, we focused on patients with IHD-DM and observed the changes of the EV-carried miRNAs spectrum to provide a novel biomarker for the diagnosis of IHD-DM.

In the current study, we analyzed the EV-carried miRNAs spectrum from plasma obtained from IHD-DM patients and healthy individuals and identified circulating EV-carried microRNAs (miRNAs) as novel diagnostic biomarkers of IHD-DM.

## Materials and Methods

The study was approved by the ethics committee of the First Affiliated Hospital of Zhengzhou University and all subjects gave informed consent before participation in the study.

### Patients and Sample Collection

This study includes a discovery set (*n* = 12) and a validation set (*n* = 40), involved IHD-DM patients (*n* = 32), and age- and gender-matched healthy volunteers served as controls (*n* = 20). The diagnostic criteria of DM were based on hemoglobin A1c (HbA1c) or FPG or the oral glucose tolerance test (OGTT) (18). IHD was defined by the symptoms and invasive coronary angiography (19). The exclusion criteria included acute myocardial infarction, type 1 diabetes, rheumatic heart disease, severe heart failure, severe heart valve disease, recent (<3 months) major surgical procedures or trauma, serious dysfunction of the liver or kidney, malignant tumor, and infectious disease. Fasting blood samples were collected in ethylenediaminetetraacetic acid tubes from healthy controls and IHD-DM patients after signing informed consent, which was obtained following the declaration of Helsinki. The blood sample was centrifuged at 3,000 rpm for 15 min, and the supernatant containing plasma was collected and stored in a refrigerator at −80°C for further analyses. None of the clinical parameters other than the occurrence of T2DM and HbA1c concentration were significantly different between the two groups.

### Isolation of Plasma-Derived EVs

ExoQuick Exosome Precipitation Solution (System Biosciences, Palo Alto, CA, United States) was used to extract EVs from the supernatant containing plasma according to the manufacturer's instructions. In brief, plasma samples were thawed in a water bath at 25°C, then the 1,600 μL supernatant was used to obtain EVs *via* adding 403.2 μL ExoQuick reagent and incubated for 1 h at 4°C. After centrifugation at 4,000 rpm for 30 min and 4,000 rpm for 5 min, the pelleted EVs were resuspended by phosphate buffer solution (PBS), and the EVs solution was stored at −80°C.

### miRNA Extraction From EVs

microRNAs were isolated from EVs using the TRIzol method according to the manufacturer's instructions. In brief, we added 800 μL Trizol reagent (Invitrogen, Waltham, MA, United States) to the 1,600 μL buffer containing the purified EVs. After mixing and dissolving for 5 min, we centrifuged the tubes at 12,000 rpm for 10 min to obtain the supernatant and then placed it at 4°C for 10 min. We then added 200 μL chloroform to the supernatant, placed it at 4°C for 10 min, and centrifuged it at 12,000 rpm for 10 min to obtain the supernatant. Then, the same volume of isopropanol and glycogen was added to the supernatant, and the mixture was stored overnight at −20°C. The next day, the supernatant was discarded after centrifugation at 12,000 rpm for 10 min. Finally, we added 1,000 μL of 70% ethanol and the mixture was centrifuged at 12,000 rpm for 10 min. The quantity and quality of the EV-carried miRNA were further analyzed using Nanodrop (Thermo Fisher Scientific, United States). Purified EV-carried miRNAs were used for further RNA sequencing analysis.

### Small RNA Library Construction and Sequencing

The EV-carried miRNA library was constructed using TruSeq Small RNA Sample Prep Kits (Illumina, San Diego, CA, United States) according to the manufacturer's instructions. After obtaining the raw data, the Cutadapt software was used to cut off the connectors at both ends of the reads and retain the reads whose length after pruning was longer than that of 17 nucleotides. The FANse3 ultra-high precision sequence alignment algorithm was used to compare the reads obtained from the sequencing of each sample with the reference sequence of the species (Human mature miRNA, miRBase 22.1).

### Immunoblot Analysis of EVs Markers

Extracellular vesicle proteins were quantified using the bicinchoninic acid (BCA) assay. Then, 25 μg of protein was put on 10% gradient SDS-PAGE gels and transferred onto polyvinylidene fluoride. And then the polyvinylidene fluoride was blocked with 5% skimmed milk and subsequently incubated overnight at 4°C with specific primary antibodies such as anti-CD63 antibody (abcam, ab134045), and anti-CD81 antibody (abcam, ab109201). After being washed four times, the polyvinylidene fluoride was incubated with a specific secondary antibody for 1 h, and proteins were detected using the enhanced chemiluminescence method with horseradish peroxidase kit (Thermo Fisher Scientific, Waltham, MA, United States) and visualized by a gel imaging system (AI600, GE Healthcare, United States).

### Transmission Electron Microscope

Extracellular vesicles were visualized using a JEOL-1230 transmission electron microscope. In brief, 5 μL of EVs were fixed in 4% paraformaldehyde and deposited onto formvar-carbon on microscopy grids. Then, the grids were washed with 100 μL PBS, followed by 50 μL 1% glutaraldehyde for 5 min, and then eight times with 100 μL ddH_2_O each for 2 min. Finally, the grids were stained with uranium oxalate at a pH of 7 for 5 min, and then with methylcellulose for 10 min. After drying the grids, the microscope images were captured at 80 kV.

### Nanoparticle Tracking Analysis

We measured the particle size and concentration of EVs were measured using nanoparticle tracking analysis (NTA) with ZetaView PMX 110 (Particle Metrix, Munich, Germany) and corresponding software ZetaView 8.04.02. Isolated EV samples were appropriately diluted using PBS to measure the particle size and concentration. NTA measurement was recorded and analyzed at 11 distinct positions. The ZetaView system was calibrated using 110 nm polystyrene particles. The temperature was maintained around 23°C to 30°C.

### Quantitative Real-Time PCR

To validate the data of small RNA sequencing, we performed quantitative real-time PCR (qRT-PCR) analysis of selected miRNAs. Plasma samples from 14 healthy controls and 26 patients with IHD-DM were collected. First, EV-carried miRNAs were reverse transcribed into cDNA by the PrimeScript RT reagent Kit (TaKaRa, Otsu, Shiga, Japan) according to the manufacturer's instructions. Then, synthesized cDNA was used as a template for all the qRT-PCR reactions performed with SYBR Premix Ex Taq (TaKaRa, Otsu, Shiga, Japan). Melting curve analysis was used to confirm the specificity of the amplification reactions. Relative miRNA quantification on the validation set was calculated by the 2^−Δ*ΔCT*^ method, that is, the normalized gene expression (2^−Δ*CT*^) in the sample A divided by the normalized gene expression (2^−Δ*CT*^) in sample B. The ΔCT was equal to the CT values of the target gene minus the CT values of the reference gene. The primers that were used to determine the expression levels of miRNAs are shown in Supplementary Table 1.

### Data Analysis

After preprocessing and quality control of the RNA-seq files, the miRNA mapping results were normalized using transcripts per kilobase of exon model per million mapped reads (TPMs). MiRNAs with an average read count of more than five TPMs were considered for further analysis. The pairwise differences in miRNA expression levels between the healthy controls group and IHD-DM patients group were examined using the edgeR RNA-seq differential analysis method. To be considered to have a significant concentration change, miRNAs required a fold change >2 [or a log_2_ (fold change) > ±1)]with *P* < 0.05. Continuous variables were presented as the mean ± standard deviation, and an Unpaired two-tailed *t*-test (normally distributed data) or Mann–Whitney *U* test (non-normally distributed data) was performed for two-group comparisons. The receiver–operating characteristic (ROC) curve was used to assess the diagnostic efficacy of the EV-carried miRNAs selected for the validation set.

## Results

### Characterization of Plasma-Derived EVs

**Figure 1:**
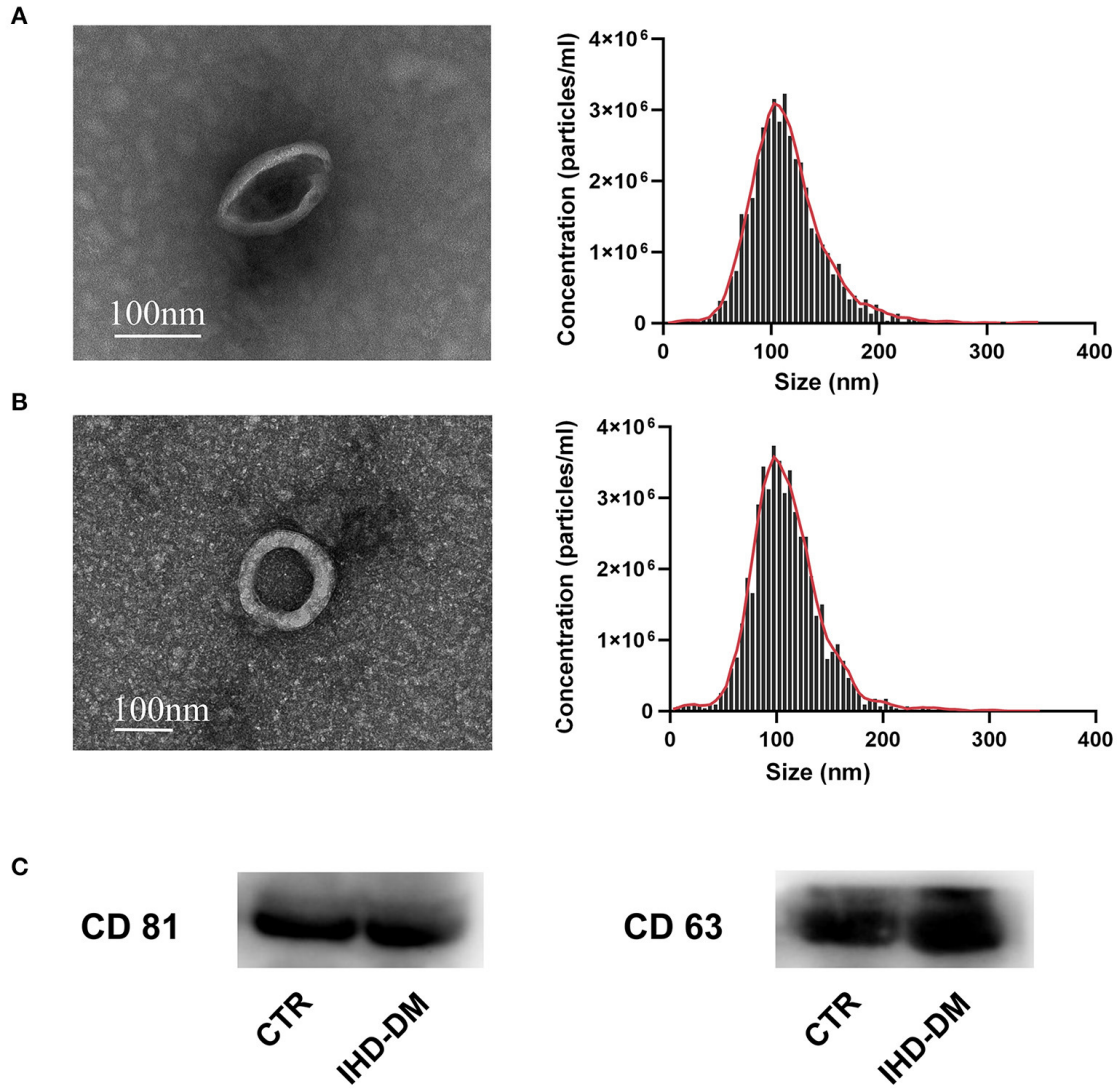
Human sourced plasma-derived EVs were collected and characterized. **(A)** Representative transmission electron microscopy (TEM) images and the nanoparticle tracking analysis (NTA) of plasma-derived EVs from healthy controls. **(B)** Representative TEM and NTA of plasma-derived EVs from IHD-DM patients. **(C)** Representative immunoblot images show enrichment of EV/exosomal markers CD63 and CD81 in plasma-derived EVs. IHD-DM: ischemic heart disease patients with diabetes mellitus; CTR: healthy controls.

Transmission electron microscope analysis revealed the cup-shape morphology of EVs from the healthy controls and IHD-DM patients, and the NTA analysis indicated that plasma-derived EVs from both groups ranged from 50 to 150 nm (Figures 1A,B). Immunoblotting exhibited EV markers such as CD63 and CD81 in the plasma-derived EVs (Figure 1C and Supplementary Figure 1).

### Identification of Differentially Expressed EV-Carried miRNAs in Healthy Controls and IHD-DM Patients

**Figure 2:**
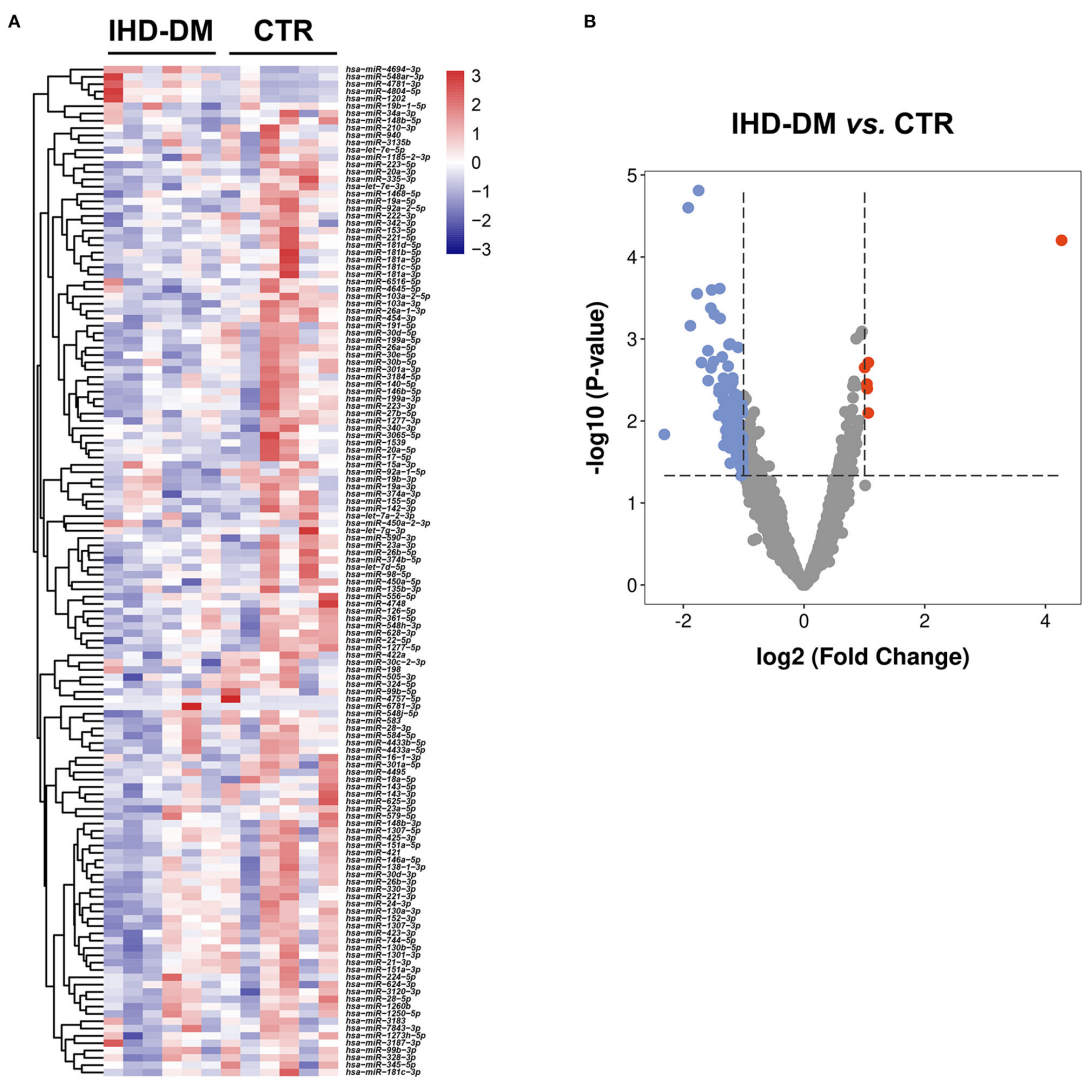
Cluster analysis of differentially expressed EV-carried miRNAs between IHD-DM and healthy controls. **(A)** Heatmap of differentially expressed EV-carried miRNAs between IHD-DM patients and healthy controls. **(B)** Volcano plots of EV-carried miRNAs expression levels in plasma of IHD-DM and healthy controls. *x*- and *y*-axes show estimated expression difference measured in log_2_ (fold change) and the significance of the expression difference measured in -log_10_ (*P*-value), respectively. Vertical lines referred to a two-fold difference in expression between the two groups.

The average reads of EVs-miRNAs from healthy controls and IHD-DM patients were 12.58 ± 1.2 and 8.73 ± 1.29 million reads, respectively. Hierarchical clustering showed that the EV-carried miRNA signature distinguished IHD-DM from healthy controls (Figure 2A). We found that 138 (6 up-regulated and 132 down-regulated) miRNAs were significantly modulated greater than or equal to two-fold in IHD-DM compared with healthy controls (Figure 2B).

### Validation of EV-Carried miRNAs in Healthy Controls and IHD-DM Patients

**Figure 3:**
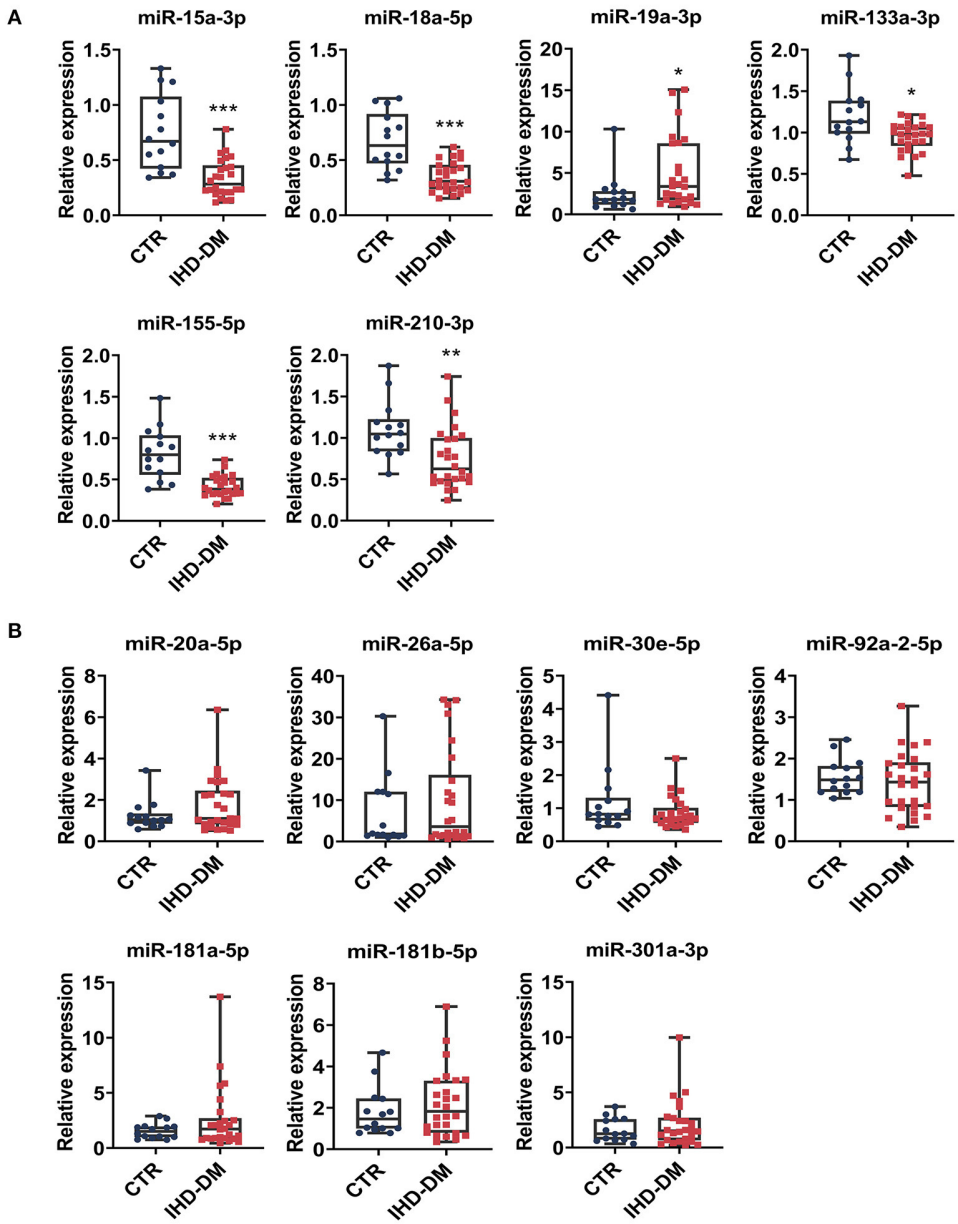
Validation of differentially expressed EV-carried miRNAs between healthy controls and IHD-DM patients. **(A)** EV-carried miRNAs including miR-15a-3p, miR-18a-5p, miR-19a-3p, miR-133a-3p, miR-155-5p, and miR-210-3p are differentially expressed between the IHD-DM patients and healthy controls. **(B)** miRNAs such as miR-20a-5p, miR-26a-5p, miR-30e-5p, miR-92a-2-5p, miR-181a-5p, miR-181b-5p, and miR-301a-3p were not statistically significant between the two groups. *P < 0.05, **P < 0.01, and ***P < 0.001 indicates significance compared with the control group.

We selected 13 circulating EV-miRNAs with which to further investigate the differential expression between the IHD-DM and healthy controls group via qRT-PCR. It could be found that the levels of miR-15a-3p, miR-18a-5p, miR-133a-3p, miR-155-5p, and miR-210-3p were significantly down-regulated in IHD-DM patients compared to healthy controls (Figure 3A). In contrast, the expression of miR-19a-3p was up-regulated in the IHD-DM patients when compared with those in the healthy individuals (Figure 3A). The other seven miRNAs, namely miR-20a-5p, miR-26a-5p, miR-30e-5p, miR-92a-2-5p, miR-181a-5p, miR-181b-5p, and miR-301a-3p, did not have statistically different expressions between the two groups (Figure 3B).

### EV-Carried miRNAs as Diagnostic Biomarkers for IHD-DM

**Figure 4:**
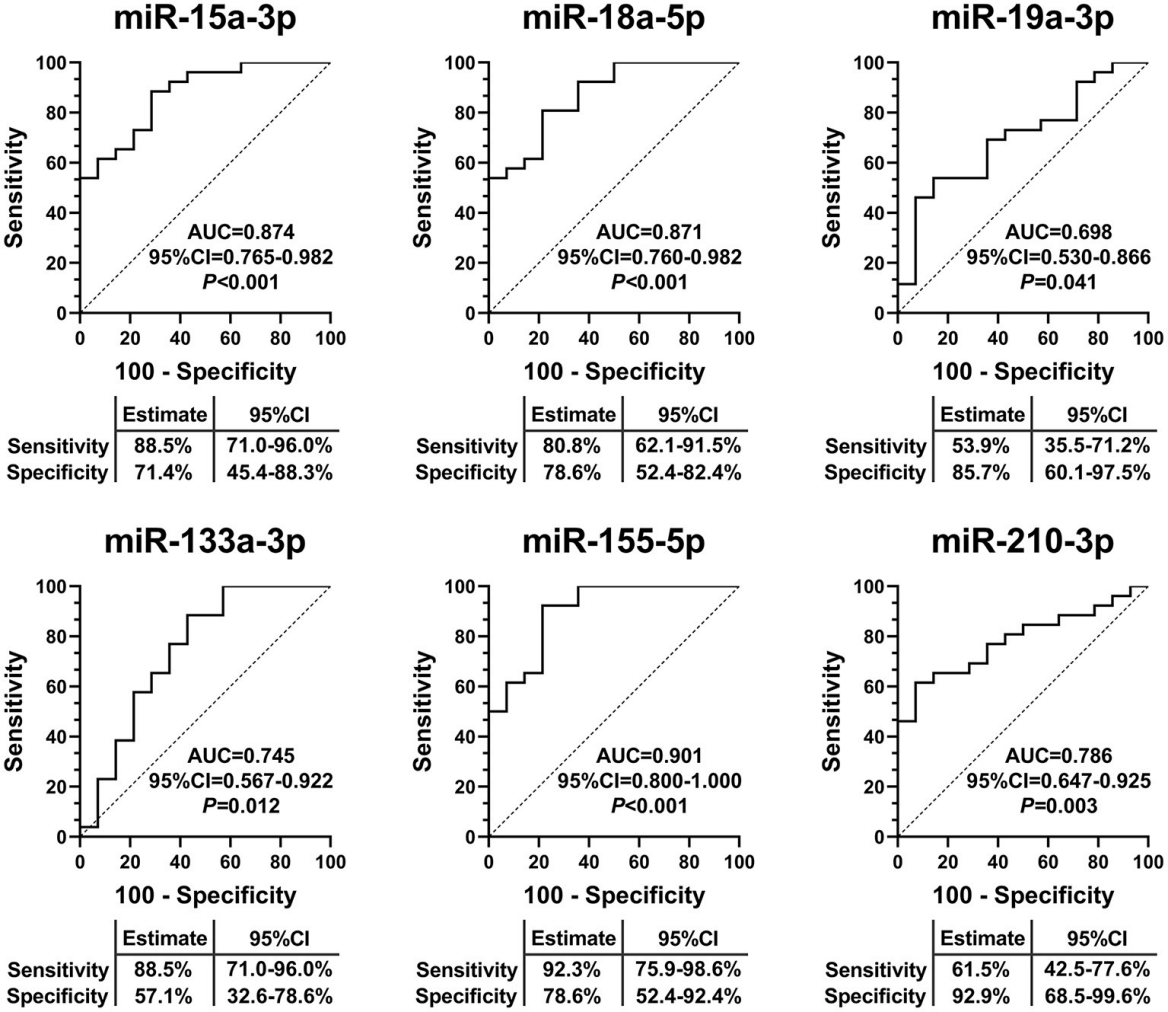
EV-carried miRNAs as useful biomarkers to distinguish IHD-DM from healthy controls. ROC curve analyses displayed EV-carried miR-15a-3p, miR-18a-5p, miR-19a-3p, miR-133a-3p, miR-155-5p, and miR-210-3p as useful biomarkers to discriminate IHD-DM from healthy controls. *P* < 0.05 was considered statistically significant. AUC: area under the curve.

To assess the specificity and sensitivity of EV-carried miRNAs for the diagnosis of IHD-DM, ROC curve analysis was performed on the validation set. The area under the curve (AUC) of miRNAs was used to quantify the diagnostic efficacy of these miRNAs. The ROC curve analysis results showed that miR-15a-3p (0.874, 95%CI: 0.765-0.982), miR-18a-5p (0.871, 95%CI: 0.760-0.982), miR-19a-3p (0.698, 95%CI: 0.530-0.866), miR-133a-3p (0.745, 95%CI: 0.567-0.922), miR-155-5p (0.901, 95%CI: 0.800-1.000), and miR-210-3p (0.786, 95%CI: 0.647-0.925) were able to discriminate IHD-DM from healthy controls (Figure 4).

## Discussion

In the present study, we identified 138 differentially expressed EV-carried miRNAs between IHD-DM patients and healthy controls through small RNA sequencing. Selected EV-carried miRNAs (miR-15a-3p, miR-18a-5p, miR-19a-3p, miR-20a-5p, miR-26a-5p, miR-30e-5p, miR-92a-2-5p, miR-133a-3p, miR-155a-5p, miR-181a-5p, miR-181b-5p, miR-210-3p, and miR-301a-3p) were further verified in a larger population through qRT-PCR technology. In comparison to healthy controls, miR-15a-3p, miR-18a-5p, miR-133a-3p, miR-155-5p, and miR-210-3p were down-regulated in IHD-DM patients, whereas miR-19a-3p was up-regulated in the IHD-DM patients (Figure 3). Among the numerous differentially expressed EV-carried miRNAs, miR-155-5p, miR-15a-3p, and miR-18a-5p are excellent biomarkers for the diagnosis of IHD-DM with the AUC of 0.901, 0.874, and 0.871, respectively (Figure 4).

**Figure 5:**
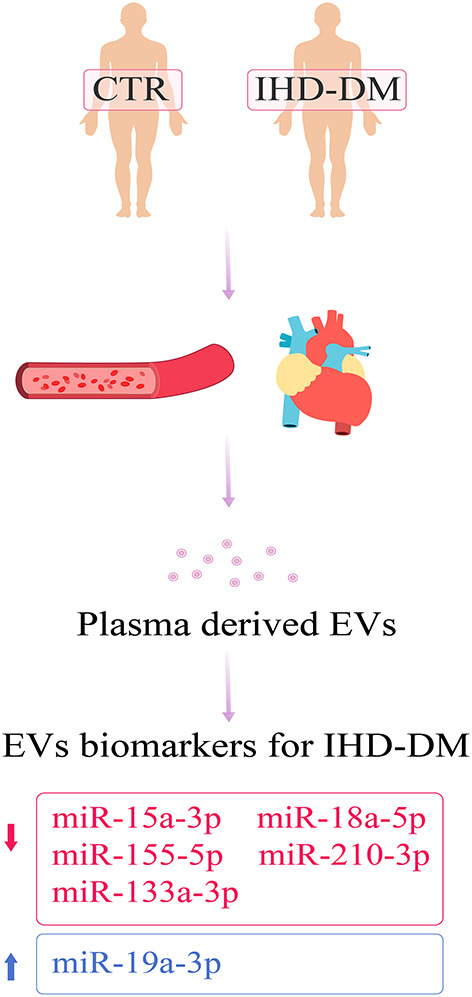
Schematic summary for the identified EVs-derived miRNA biomarkers for IHD-DM patients.

Previous studies have suggested that multiple mechanisms are involved in the pathological progression of diabetic angiopathy, including hyperglycemia, insulin resistance, hyperlipidemia, inflammation, reactive oxygen species, endothelial dysfunction, hypercoagulability, and vascular calcification (20, 21). Further studies showed that dysregulation of EV-carried miRNAs plays an important role in the process of diabetic heart disease (12). Stepień et al. demonstrated that circulating EVs-carried miRNAs are significantly different between patients with T2DM and healthy controls, suggesting that dysregulation of EV-carried miRNAs is associated with the occurrence of vascular complications in patients with T2DM (22). Thus, EV-carried miRNAs are considered to reflect the pathophysiological state of the disease (23). In the present study, we screened and identified the differences in EV-carried miRNA cargoes between healthy controls and IHD-DM patients (Figure 5).

Accumulating evidence has demonstrated that diabetes-mediated myocardial fibrosis is an important cause of myocardial stiffness and diastolic dysfunction in the diabetic heart (24). Rawal et al. demonstrated that the miR-15 family is involved in fibrotic remodeling of the diabetic heart (25). Furthermore, they found a significant down-regulation of miR-15a and miR-15b in myocardial tissue from diabetic patients complicated with cardiovascular disease (25). Additionally, Geng et al. investigated the expression of miR-18a-5p in human aortic valvular endothelial cells and found the overexpression of miR-18a-5p could down-regulate Notch2 expression and subsequently suppress endothelial–mesenchymal transition, to inhibit myocardial fibrosis (26). The results of the present study are in agreement with the above research, showing that EV-carried miR-15a-3p and miR-18a-5p in plasma are significantly down-regulated in IHD-DM patients compared to healthy controls. Interestingly, previous studies showed that miR-19a-3p could significantly improve myocardial fibrosis by inhibiting autophagy-mediated fibrogenesis through targeting TGF-β R II mRNA (27), while Zhu et al. suggested an up-regulation of circulating miR-19a-3p in patients with gestational diabetes mellitus (28). Our current study also found a significant up-regulation of EV-carried miR-19a-3p in IHD-DM patients compared to healthy controls. Therefore, the role of EV-carried miR-19a-3p in diabetic heart disease needs to be further studied.

Diabetes-related vascular inflammation plays an important role in accelerating atherosclerosis formation and plaque rupture, leading to severe acute cardiovascular events (29). Prior studies have demonstrated that a variety of circulating miRNAs such as miR-181b-5p, miR-210-3p, miR-19a, and miR-181a-5p, are involved in vascular inflammation (30–33). Chen et al. suggested that the level of miR-19a is elevated in atherosclerosis-prone ascending aortic wall tissues, which promoted vascular inflammation and foam cell formation by targeting HBP-1 in atherogenesis (32). Qiao et al. demonstrated that miR-210-3p can reduce lipid accumulation and inflammatory response in ox-LDL-induced macrophages by inhibiting the expression of CD36 and NF-κB (33). In the present study, we revealed that the expression of these pro-inflammatory and anti-inflammatory EVs-carried miRNAs were significantly dysregulated, which supports the idea that diabetic status could change the expression of specific inflammatory miRNAs in plasma EVs that is possible to promote systemic or vascular inflammation.

Poor angiogenesis or calcification is also a critical factor worsening the prognosis of diabetic angiopathy. MiR-155-5p is considered to suppress anti-inflammatory signaling in macrophages and participate in the regulation of neovascularization (34, 35). Previous studies on miR-155-5p demonstrated a significant down-regulation in peripheral blood mononuclear cells and whole blood from patients with diabetes (36). Additionally, the expression level of circulating miR-133a-3p is also shown to be significantly down-regulated in patients with diabetes compared with controls (37), which results in the inhibition of human vascular smooth muscle cell proliferation and the induction of cell apoptosis *via* matrix metalloprotein-9 (MMP-9) (38). In the present study, we found that EV-carried miR-155-5p and miR-133a-3p are significantly down-regulated in IHD-DM patients compared to healthy controls.

Moreover, our current work also indicated that there were no significant difference in EV-carried miR-20a-5p, miR-26a-5p, miR-30e-5p, miR-92a-2-5p, miR-181a-5p, miR-181b-5p, or miR-301a-3p in plasma between healthy controls and IHD-DM patients. Concerning miR-181a-5p, previous studies suggested that miR-181a-5p could cooperate with miRNA-181a-3p to restrict vascular inflammation and atherosclerosis, and showed that the level of miR-181a-5p level was significantly reduced in the aorta plaque and plasma (30). However, the expression level of miR-181a-5p in diabetic patients is still debated. Lozano-Bartolomé et al. showed that the expression of miR-181a-5p is reduced in adipose tissue from diabetic subjects (38), whereas other studies showed a significant up-regulation in serum or adipose tissue from diabetic patients (39). In our study, no significant difference in EV-carried miR-181a-5p in plasma was found between healthy controls and IHD-DM patients. Therefore, future research is necessary to further confirm the expression of these EV-carried miRNA in diabetic patients with cardiovascular disease.

There are several limitations to the present study. First, the plasma was collected from patients in a single clinical center, which weakens the external validity required to support widespread changes in practice (40). Hence, a multicenter large sample study should be conducted in the future. Furthermore, we extracted the circulating EVs only through the ExoQuick methods, but not the combination of the ultracentrifugation method and the ExoQuick method. In addition, the present study systematically explored the EV-carried miRNA expression profiles of IHD-DM patients. However, the detailed pathogenesis of these differentially expressed EV-carried miRNA should be explored in future research.

## Conclusion

The present study systematically reveals the differential expression profile of plasma-derived EV-carried miRNAs through small RNA-sequencing analysis between IHD-DM patients and healthy controls. And these EV-carried miRNAs were further verified in a larger population. Our data suggest that a few of the EVs-carried miRNAs found to be differentially expressed between IHD-DM patients and healthy controls have the potential to be novel circulating EV-carried miRNA biomarkers.

## Data Availability Statement

The datasets presented in this study are deposited in the NCBI Sequence Read Archive repository, accession number: PRJNA792080.

## Ethics Statement

The studies involving human participants were reviewed and approved by Ethics Committee of the First Affiliated Hospital of Zhengzhou University. The patients/participants provided their written informed consent to participate in this study.

## Author Contributions

JT and JinZ: conception and design. LZ, JiaZ, ZQ, NL, and ZZ: analysis and interpretation of data. LZ and JiaZ: drafting the article. YL and YX: critically revising the article. JT, LZ, and JinZ: reviewing submitted version of manuscript. All authors contributed to the article and approved the submitted version.

## Funding

This work was supported by the National Natural Science Foundation of China (Nos. 81800267, 81870328, U2004203, and 82170281), the Henan Medical Science and Technology Joint Building Program (No. 2018020002), the Henan Thousand Talents Program (No. ZYQR201912131), the Henan Province Youth Talent Promoting Project (No. 2020HYTP051), and the Excellent Youth Science Foundation of Henan Province (No. 202300410362).

## Conflict of Interest

The authors declare that the research was conducted in the absence of any commercial or financial relationships that could be construed as a potential conflict of interest.

## Publisher's Note

All claims expressed in this article are solely those of the authors and do not necessarily represent those of their affiliated organizations, or those of the publisher, the editors and the reviewers. Any product that may be evaluated in this article, or claim that may be made by its manufacturer, is not guaranteed or endorsed by the publisher.
