# Supplementary material for: Diagnostic and Predictive Values of Circulating Extracellular Vesicle-Carried microRNAs in Ischemic Heart Disease Patients With Type 2 Diabetes Mellitus
Source: Front Cardiovasc Med. 2022 Feb 28;9:813310. doi: 10.3389/fcvm.2022.813310 (PMC8918773; doi:10.3389/fcvm.2022.813310)
Supplement: Supplementary file 1 [file Data_Sheet_1.docx]

### Supplementary Material

### Supplementary Table 1. Primer sequences of selected miRNAs in the study

| miRNA ID | Primer sequence |
| --- | --- |
| hsa-miR-210-3p | 5’-AACAAGCTGTGCGTGTGACA-3’ |
| hsa-mir-155-5p | 5’-AAGCGACCTTAATGCTAATCGTGA-3’ |
| hsa-miR-133a-3p | 5’-AACACGCTTTGGTCCCCTTC-3’ |
| hsa-miR-15a-3p | 5’-AACACGCCAGGCCATATTGTG-3’ |
| hsa-miR-181a-5p | 5’-AACACGCAACATTCAACGCTG-3’ |
| hsa-miR-181b-5p | 5’-AAGCGACCAACATTCATTGCTGT-3’ |
| hsa-miR-30e-5p | 5’-AGCCAGCGTGTAAACATCCTTG-3’ |
| hsa-miR-18a-5p | 5’-AAGCGACCTAAGGTGCATCTAGT-3’ |
| hsa-miR-26a-5p | 5’-AGCCAGCGTTCAAGTAATCCAG-3’ |
| hsa-miR-20a-5p | 5’-ATGCGCGCTAAAGTGCTTATAGT-3’ |
| hsa-miR-19a-3p | 5’-AACACGCTGTGCAAATCTATGC-3’ |
| hsa-miR-301a-3p | 5’-AAGCGACCCAGTGCAATAGTATT-3’ |
| hsa-miR-92a-2-5p | 5’-AACAGTGGGGTGGGGATTTGT-3’ |


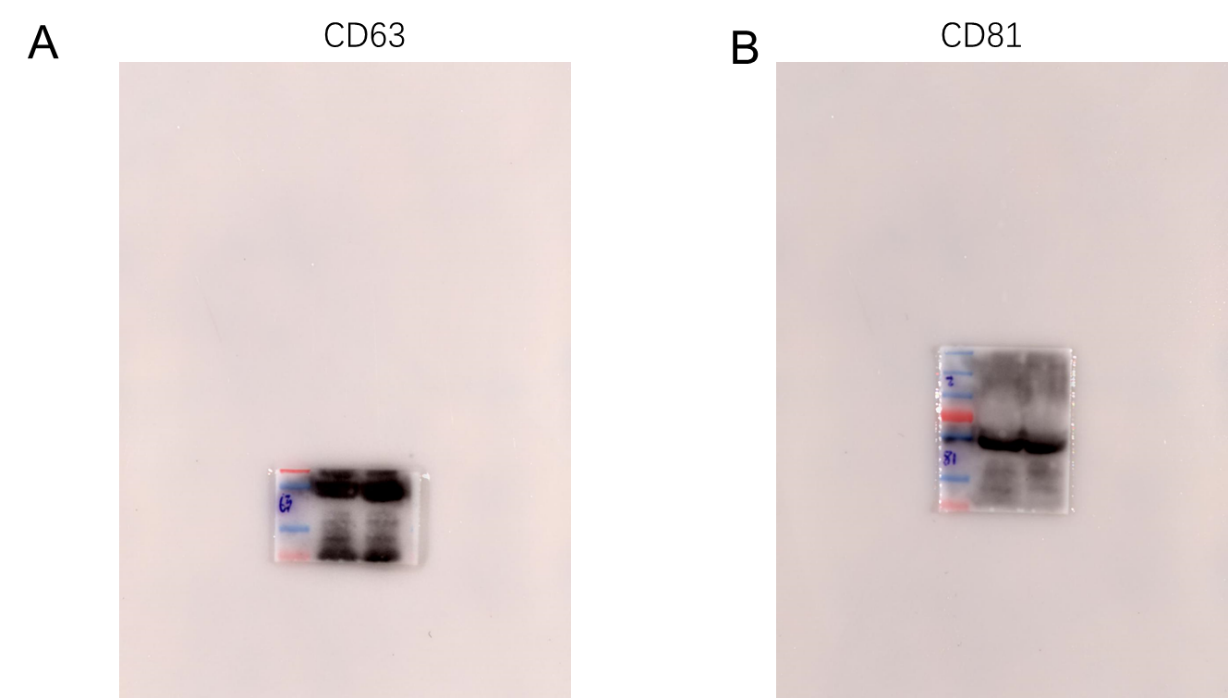


Supplementary Figure 1. Full western blot gels of extracellular vesicle (EV)/exosomal markers CD63 and CD81 in plasma-derived EVs.
